# Adsorption of Chiral [5]-Aza[5]helicenes on DNA Can Modify Its Hydrophilicity and Affect Its Chiral Architecture: A Molecular Dynamics Study

**DOI:** 10.3390/ma13215031

**Published:** 2020-11-07

**Authors:** Giuseppina Raffaini, Andrea Mele, Tullio Caronna

**Affiliations:** 1 Department of Chemistry, Materials and Chemical Engineering “Giulio Natta”, Politecnico di Milano, Piazza L. Da Vinci 32, 20131 Milano, Italy; andrea.mele@polimi.it; 2 INSTM, National Consortium of Materials Science and Technology, Local Unit Politecnico di Milano, 20131 Milano, Italy; 3 Dipartimento di Ingegneria e Scienze Applicate, Università degli Studi di Bergamo, 24044 Bergamo, Italy; tullio.caronna@fastwebnet.it

**Keywords:** chirality, intrinsic chirality, helicenes, structural selectivity, adsorption, DNA recognition, molecular dynamics simulations, racemic mixture, cholesteric liquid crystals

## Abstract

Helicenes are interesting chiral molecules without asymmetric carbon atoms but with intrinsic chirality. Functionalized 5-Aza[5]helicenes can form non-covalent complexes with anticancer drugs and therefore be potential carriers. The paper highlights the different structural selectivity for DNA binding for two enantiopure compounds and the influence of concentration on their adsorption and self-aggregation process. In this theoretical study based on atomistic molecular dynamics simulations the interaction between (*M*)- and (*P*)-5-Aza[5]helicenes with double helix B-DNA is investigated. At first the interaction of single pure enantiomer with DNA is studied, in order to find the preferred site of interaction at the major or minor groove. Afterwards, the interaction of the enantiomers at different concentrations was investigated considering both competitive adsorption on DNA and possible helicenes self-aggregation. Therefore, racemic mixtures were studied. The helicenes studied are able to bind DNA modulating or locally modifying its hydrophilic surface into hydrophobic after adsorption of the first helicene layer partially covering the negative charge of DNA at high concentration. The (*P*)-enantiomer shows a preferential binding affinity of DNA helical structure even during competitive adsorption in the racemic mixtures. These DNA/helicenes non-covalent complexes exhibit a more hydrophobic exposed surface and after self-aggregation a partially hidden DNA chiral architecture to the biological environment.

## 1. Introduction

The DNA can be considered as a chiral helical polymer in a double-stranded arrangement that generally adopts right-handed helices under physiological conditions, B-DNA, one of three biologically active double-helical structures with right-handed double-helical structure, and it may change to left-handed, Z-DNA, one of three biologically active double-helical structures with left-handed double-helical structure first discovered by Robert Wells and colleagues [1], depending on the sequence and medium conditions (B–Z transition). The study of the interaction between DNA structure and chiral molecules in which the chirality is due to chiral architecture not for asymmetric carbon atoms in the structure is increasingly important.

Chiral molecules are known to intercalate in double-stranded DNA or to adsorb on biomolecule grooves leading to the formation of complexes. The chiral helicenes can interact with double strand DNA thus influencing the mechanism in which it is involved and modifying its function in physiological processes, desirable for the treatment of diseased cells. In general, molecules interact with DNA by intercalation if they are small planar aromatic molecules, or by binding to the minor or major grooves of DNA due to favorable interaction and structural selectivity [2,3,4,5].

Helicenes are chiral hydrophobic polycyclic compounds formed by a variable number of ortho-fused benzene or other aromatic rings with a helical backbone, without asymmetric carbon atoms but with intrinsic chirality [6,7,8,9,10]. These chiral molecules interacting with π–π interactions are useful as building blocks of materials important in optoelectronic applications, for their chelating properties [7] and, interestingly, when functionalized, they can form non-covalent complexes with anticancer drugs and therefore be potential carriers. Hence, the range of possible applications of helicenes is very wide such as liquid crystals, sensors, dyes, asymmetric synthesis, molecular recognition, polymer synthesis, materials science and biomedical applications [11,12,13,14,15,16,17,18]. Helicenes are an interesting family of molecules with intrinsic chirality that can exhibit chiral selectivity and structural selectivity for binding to DNA and it can discriminate between B- and Z-DNA [5,19]. These hydrophobic chiral molecules self-aggregate due to hydrophobic interactions, forming aggregates that look like liquid crystal systems with preferred orientation after self-aggregation. Self-aggregation also occurs on the chiral structure of the DNA at higher concentration due to the favorable interaction with the first adsorbed helicene layer. Sometimes the helicenes can also separate the crystalline nanophase [20]. Therefore, helicenes are possible promising building blocks for new materials.

Monomeric or linked polyamides in the hairpin motif have been used for binding affinity to B-DNA of the *R* enantiomer that can in general strongly enhance the interaction [2,3], also suggesting that transcription inhibition of selected gene promoters can be achieved with polyamides well interacting with DNA [4]. Using spectroscopic techniques, including time-resolved fluorescence, fluorescence anisotropy and linear dichroism (LD), Kel et al. reported that [4]helicene derivatives, for example, show chiral selectivity in binding to double-stranded DNA being promising candidates for biomedical sensing [21]. There are some chiral ligands capable of binding *Z*-DNA and also converting B- to *Z*-DNA. Then there was more attention focused on binding relatively small molecules to specific DNA structures to inhibit the biological functions in which these particular structures participate. For example, the (*P*)-1,14-dimethyl[5]helicene ligand preferentially binds to B-DNA, whereas its (*M*)-enantiomer shows a higher affinity to Z-DNA [22,23]. Another simple helicene molecules reported by Xu et al. containing sulphur atoms in the chiral structure display structural selectivity in binding to DNA: from circular dichroism (CD) spectra the specific (*P*)-enantiomer considered selectively binds Z-DNA and it is able to convert the B-DNA conformation to Z-DNA [24]. The structural selectivity of helicenes offers a new route for the rational design of inhibitors of biological functions that may depend on the stability of DNA helices.

The synthesis and characterization of Aza-[5]helicenes bearing one or two nitrogen atoms in selected ring positions was reported for the first time by Caronna et al. [20,25,26]. Nitrogen-substituted heteroaromatic molecules are gaining increasing interest owing to the fact their complexes with transition metal ions show interesting properties in harvesting visible light and re-emitting it at a wavelength that depends on the metal ion used. The theoretical study based on molecular mechanics (MM) and molecular dynamics (MD) simulations can be useful to atomistically describe the specific helicene and the DNA structure and therefore to better understand the interaction with these chiral molecules. In this work, the adsorption process of (*M*)-5-Aza[5]helicene, (*M*)-HA, and (*P*)-5-Aza[5]helicene, (*P*)-HA, on double-stranded B-DNA were studied both in terms of interaction strength, interaction geometry at a specific interaction site, minor groove or major groove of B-DNA. First the interaction of single pure enantiomers on the chiral surface of B-DNA was investigated, then the adsorption process at two different finite concentrations, a low and a high concentration were studied, finally racemic mixtures were also considered. Interestingly, the adsorption process can be affected by the helicene concentration. The competitive adsorption process on double-stranded DNA is reported adopting the same simulation protocols proposed in previous work on the theoretical study of non-covalent interactions in the formation of inclusion cyclodextrin complexes [27,28,29] and the adsorption of proteins on biomaterials surfaces influenced also by the topography, in particular the curvature of carbon nanotubes also having different chirality [30,31]. Furthermore, the competitive adsorption [32] and the self-aggregation process of systems that can spontaneously form aggregates [33] and the importance of drug concentration in nanocarriers devices [34] can be considered atomistically using MM and MD simulations. Using the same theoretical methods adopted in previous work, the aggregation of enantiopure compound or racemic mixture of 5-Aza[5]helicenes, at low and high concentrations, was also studied. In the present theoretical work the (*P*)-5-Aza[5]helicene displays a slightly larger affinity for the B-DNA fragment than the (*M*)-enantiomer, but it will always be important to study the interactions and possible conformational changes of chiral systems induced by other chiral molecules. Here the theoretical study of 5-Aza[5]helicene self-aggregation process using MM and MD methods is also reported for a comparison in order to understand the strong effect of concentration observed in our study.

## 2. Materials and Methods

Using theoretical methods based on molecular mechanics (MM) and molecular dynamics (MD) simulations, the interaction between helicenes and double-stranded DNA was investigated. All MM and MD simulations were performed using the consistent valence force field CVFF [35,36,37,38,39,40], the Materials Studio and InsightII/Discover program packages [41]. The simulation protocol proposed in previous work [27,28,29], first involved an initial energy minimization of the system, then the MD run until the equilibrium state was achieved and finally the optimization of saved configurations assumed by the system during MD run and of the optimized geometry assumed by the system at the end of MD run. All energy minimizations in a dielectric medium, considering a distance-dependent dielectric constant of water, were performed using the conjugate gradient algorithm, the Fletcher method, minimized up to an energy gradient lower than 4 × 10^−3^ kJ mol^−1^ Å^−1^.The MD simulations last 10 ns for the study of a single enantiomer of helicene, while all MD simulations at high concentration last 20 ns. All MD simulations in an NVT ensemble (see Abbreviations) were performed in a dielectric medium at constant temperature equal to 300 K controlled through the Berendsen thermostat. Integration of the dynamical equations was carried out with the Verlet algorithm with a time step of 1 fs, and the instantaneous coordinates were periodically saved for further analysis. Within the MD runs, the total energy and the energy components in particular the potential energy and the van der Waals contribute showed a fast initial decrease, and then fluctuated around a constant value for most of the runs, indicating achievement of equilibrium. The structure of double stranded B-DNA fragment was found in the Protein Data Bank (3CRO) [42], and it was fixed during the calculations, then it is treated as a rigid body during the MD simulations. The two enantiomers were generated using the Module Builder of InsightII/Discover and finally optimized. Using a simulation protocol proposed in previous work [27,28,29], first the interaction DNA/5-Aza-[5]helicene enantiomers was studied in 1:1 stoichiometry, afterwards it was studied both at low concentration and at high concentration, considering respectively a 1:20 stoichiometry in a cubic cell of 123 Å and a 1:120 stoichiometry in a cubic cell of 141 Å using the amorphous cell module present in the Materials Studio program package. The periodic boundary conditions (PBC) and the initial random arrangements of chiral molecules in a simulation box were considered. Therefore, racemic mixtures at both low and high concentrations were investigated in order to understand the effect of the concentration and of competitive adsorption process on the chiral surface of DNA. Since helicene self-aggregation takes place and affects the adsorption process of helicenes enantiomers on double-stranded DNA, under the same theoretical conditions, starting in a random arrangement in the same simulation cell but without DNA at both low and high concentrations, the aggregation process of only the enantiopure (*M*)-HA and (*P*)-HA molecules and their racemic mixtures were also studied.

## 3. Results and Discussion

### 3.1. Self Aggregation of 5-Aza[5]helicene Molecules at Low and at High Concentration of Enantiopure Compounds and Racemic Mixtures

In order to understand the strong effect of concentration observed in our study, we first decided to check the possible self-aggregation of the 5-Aza[5]helicene.

An extended, structurally rigid, highly anisotropic shape seems to be the main criterion for liquid crystalline behavior. Many liquid crystalline materials are based on benzene rings. Different mesophases can be characterized by positional order with molecules arranged in any sort of ordered lattice, and orientational order with molecules mostly pointing in the same direction displaying one preferential direction [43,44,45].

**Figure 1:**
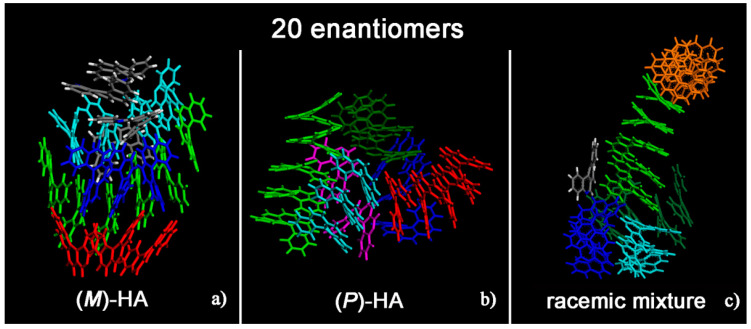
Optimized geometries at the end of the molecular dynamics (MD) run lasting 20 ns considering twenty (M)-HA molecules (**a**), twenty (*P*)-HA molecules (**b**) and the racemic mixture of twenty enantiomers (**c**). The helicenes having π–π interaction with slightly parallel aromatic rings are colored with the same color.

**Figure 2:**
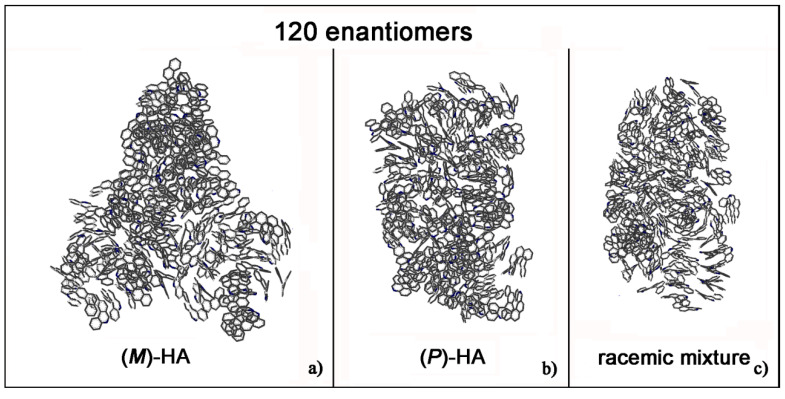
Optimized geometries at the end of MD run lasting 20 ns considering 120 (*M*)-HA (**a**), 120 (*P*)-HA (**b**) and the racemic mixture of 120 enantiomers (**c**). All atoms are in grey and the hydrogens are omitted for clarity.

Helicenes have adjacent aromatic rings in a chiral non flat structure and they are roughly discotic-like molecules. Helicenes self-aggregate because of hydrophobic interactions. The MD simulations of (*M*)- and (*P*)-HA enantiopure compounds and in a racemic mixtures at low and at high concentration, using a same simulation protocol as before, were studied. The ordering induced by both π–π interaction and their specific chiral structure leads these systems to behave like liquid crystal systems, forming aggregates exhibiting two dimensional columnar ordering with some molecules display positional ordering in a layered structure, with the molecules tilted by a finite angle with respect to the layer normal, remembering a cholesteric phase [46,47] in particular for enantiopure compounds (see Figure 1, in particular Panel a). At a low concentration different arrangements always having a preferred orientation display the same potential energy indicating that these molecules probably can form systems showing polymorphism in the solid-state. Figure 1 shows the aggregates formed considering small concentration at the end of the MD run lasting 20 ns for the enantiopure compounds (Panels a and b) and for the racemic mixture (Panel c). At high concentration different aggregates were formed as shown in Figure 2, in a particular compact structure for the (*M*)-HA forming helical distribution along three different directions (Panel a), in a regular structure with a slightly rectangular shape for the (*P*)-HA (Panel b) and of a more ellipsoidal shape for the racemic mixture (Panel c). All the different shapes probably formed the packing of these molecules in the solid state and could be important also when nanoaggregates are formed in solution. In general, for helicenes it is possible to show isomorphism, i.e., similar spatial arrangements of different molecules [48] considering for example the racemic mixture, and a different enantiopure compound can display polymorphism, i.e., different arrangements of the same molecules. In fact, during the MD run, after energy minimization of some conformations assumed by the system at room temperature, some optimized geometries display the same potential energy indicating possible different but thermodynamically equivalent ordered stable arrangement.

Lastly, recent research has shown that ellipsoidal nanoparticles (NPs) are able to extravasate from the blood vessel more effectively than spherical one [49]. Then, the study of aggregates shapes can be important to study for the possible applications of NPs in particular in a biological environment.

### 3.2. DNA/Chiral 5-Aza[5]helicene Interaction in 1:1 Stoichiometry: Importance of Chirality

**Figure 3:**
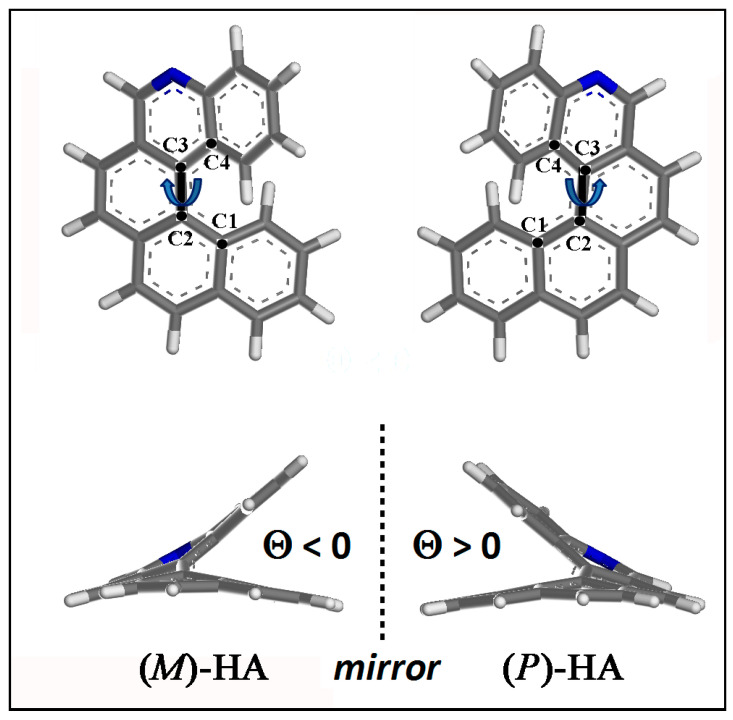
Optimized geometries of (*M*)-5-Aza[5]helicene and (*P*)-5-Aza[5]helicene. Color code: Carbon atoms are grey, the nitrogen blue and the hydrogens are white. The four carbon atoms C1, C2, C3 and C4, which describe the dihedral angles Θ for the two enantiomers, are shown.

The optimized geometries of (*M*)- and (*P*)-5-Aza[5]helicene single molecule helicene enantiomer are shown in Figure 3. The dihedral angle Θ is defined by the four carbon atoms C1, C2, C3 and C4 as reported in Figure 3: Θ is negative for (*M*)-HA being equal to −31.911° and positive for (*P*)-HA enantiomer being equal to 31.911°.

**Figure 4:**
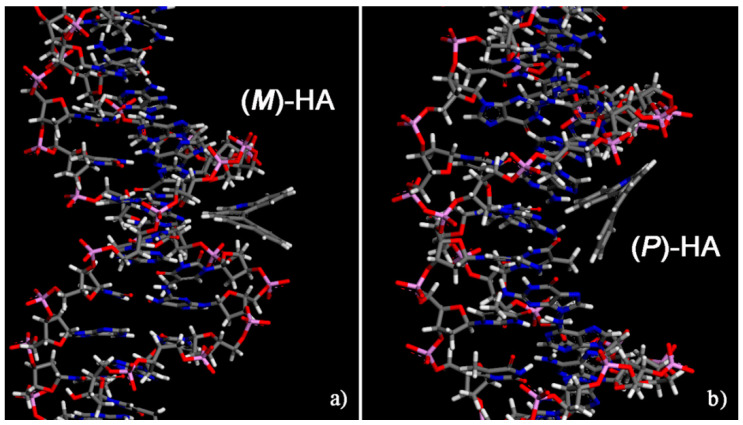
Optimized geometries of (*M*)-5-Aza[5]helicene on the left (**a**) and (*P*)-5-Aza[5]helicene on the right (**b**) after energy minimization of the conformation assumed by the system at the end of MD run lasting 10 ns. Color code: Carbon atoms are grey, the nitrogen blue, oxygens red, the phosphorus pink and hydrogens are white. The double strand DNA structure was slightly rotated to better show the two different sites of interaction.

Initially, for two different enantiomers two possible different initial geometries of interaction were considered, a geometry in which the enantiomer approaches the double strand DNA near the minor groove and a geometry in which the enantiomer approached the surface of the DNA near the major groove. The two initial geometries are reported in Figure S1 only related to the (*M*)-5-Aza[5]helicene as an example of the first step of the simulation protocol strategy adopted [27,28,29]. In the initial geometries, the single enantiomer was close to the minor groove or near the major groove of the DNA, in order to better investigate both the interaction strength and more stable geometries indicating whether a favorable interaction site is present even after the MM and MD run lasting 10 ns and final energy minimization. The most stable optimized geometries calculated for the two enantiomers at the end of MD run are shown in Figure 4; the values of the interaction energy, *E_int_*, and of the Θ dihedral angle are reported in Table 1.

The (*P*)-5-Aza[5]helicene displays a more favorable interaction with the DNA fragment than (*M*)-HA showing two similar values of interaction energies; in particular in a major groove (*P*)-HA displays a slightly more favorable interaction energy with respect to the value calculated in the minor groove site. On the contrary, the enantiomer, (*M*)-5-Aza[5]helicene exhibits better interaction strength with the minor groove, and the interaction with the major groove is less stable by approximately 10 kJ/mol. Hence, the MM and MD simulations indicate the possibility of calculating for the two enantiomers the favorable interaction geometries at a specific site of the external chiral surface exposed by DNA. No significant variations are observed in the value of the Θ dihedral angles of two enantiomers adsorbed near the minor groove and major groove of DNA fragment compared to the Θ value calculated for the isolated [5]helicene molecules.

### 3.3. DNA/5-Aza[5]helicenes Interaction: Importance of Concentration

In this section the theoretical results about the interaction of (*M*)-5-Aza[5]helicene and (*P*)-5-Aza[5]helicene molecules on the chiral surface of double stranded DNA considering two different finite concentrations were exposed.

#### 3.3.1. DNA/Chiral 5-Aza[5]helicenes Interaction at Low Concentration

**Figure 5:**
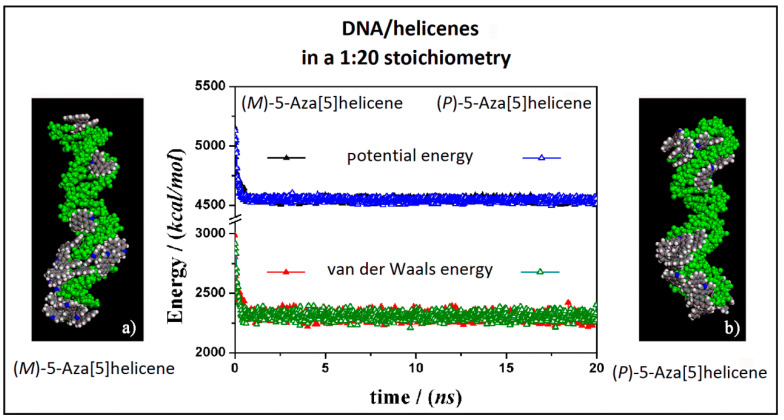
Optimized geometries of (*M*)-5-Aza[5]helicene on the left (**a**) and (*P*)-5-Aza[5]helicene on the right (**b**) after energy minimization of the conformation assumed by the system at the end of MD run considering enantiopure compounds in a 1:20 stoichiometry. In the central panel the potential energy and the van der Waals contribution calculated during the MD run for the (*M*)-HA (full symbols, black and red respectively) and the (*P*)-HA (empty symbols, blue and green respectively) are reported. All the DNA atoms are in green and the helicenes have the same color code as shown in Figure 3.

At first, the interaction between B-DNA and chiral enantiomers was considered for enantiopure compounds in a 1:20 stoichiometry, starting from initial trial geometries considering a random distribution of (*M*)-5-Aza[5]helicene and (*P*)-5-Aza[5]helicene molecules around the double strand of the DNA fragment in the center of the simulation cell as shown in Figure S2 (see the geometry in Panel a). After the MD run and energy minimizations the optimized geometries obtained are reported in Figure 5, on the left (Panel a) for (*M*)-HA enantiomer and on the right (Panel b) for the (*P*)-HA enantiomer. When performing the MD simulations for two different enantiomers the kinetics of the adsorption process on the outer surface of the DNA was very fast. After the adsorption on the DNA surface molecules of helicene were well adhered on the chiral surface, and the other molecules interacted with the π–π interaction with each other, and could move on the hydrophobic layer of the first adsorbed helicene molecule (see animation files reported below Figure S2 in SI). The values of the potential energy and the van der Waals contribution reported in Figure 5 (central panel in Figure 5) were very similar for the two different MD simulations considering two different enantiomers, displaying same kinetics of the adsorption process on DNA surface considering the enantiopure compounds.

Considering both the (*M*)-5-aza[5]helicene at a low concentration at the end of the MD run and the (*P*)-5-Aza[5]helicene at the end of the MD run a similar stability of the system was calculated. Considering the final optimized geometries it is interesting to note that the interaction geometries were slightly different (Figure 5). The optimized geometry of the (*M*)-HA enantiomer (Panel a in Figure 5) displays more molecules adhered in the minor groove a behavior just found considering the more favorable interaction of a single molecule of this enantiomer adsorbed on the DNA external surface (see Table 1). In the optimized geometry of the (*P*)-HA enantiomer (Panel b in Figure 5) the molecules were well adhered both in minor and in major groove due to an interaction energy similar to that calculated for a single molecule for these two different DNA interaction sites. Interestingly, in both cases some molecules of (*M*)-HA and (*P*)-HA interacted with the DNA surface forming a first layer of molecules that follows the helical shape of DNA and some of the other enantiomer molecules interacted with the adsorbed helicene molecules due to favorable π–π hydrophobic interactions.

**Figure 6:**
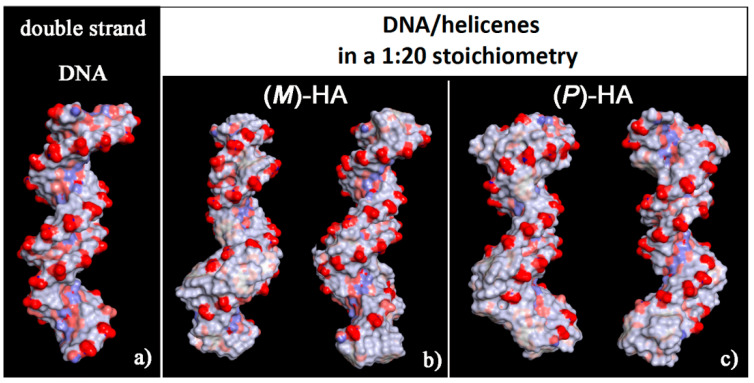
Solvent-accessible surface area colored by atoms charge for the optimized geometries of double-stranded DNA as data from Protein Data Bank on the left (**a**), of (*M*)-HA (**b**) and (*P*)-HA on the right (**c**) obtained after energy minimization of the conformation assumed by the system at the end of MD run considering enantiopure compounds in a 1:20 stoichiometry. Atoms that are partially negatively charged are in red, partially positively charged atoms in blue and apolar groups of atoms in white.

It is interesting to note that, considering the solvent-accessible surface area colored by the charge of the atoms exposed to the solvent for the two optimized geometries reported in Figure 6 it is possible to observe that this new non-covalent DNA/helicene complexes show a more hydrophobic surface, in particular in the minor groove for (*M*)-5-Aza[5]helicene, both in the minor and in the major groove for (*P*)-Aza[5]helicene, with respect to the native double-stranded DNA.

#### 3.3.2. DNA/Chiral 5-Aza[5]helicenes Interaction at High Concentration

**Figure 7:**
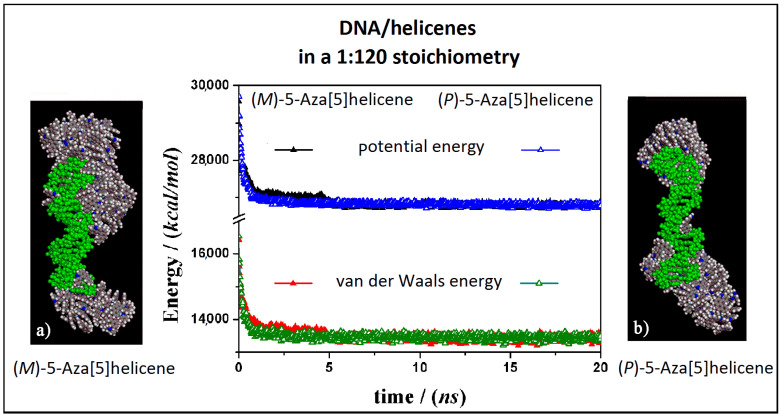
Optimized geometries of (*M*)-HA on the left (**a**) and (*P*)-HA on the right (**b**) after energy minimization of the conformation assumed by the system at the end of MD run considering enantiopure compounds in a 1:120 stoichiometry. In the central panel the potential energy and the van der Waals contribution calculated during MD run lasting 20 ns for the (*M*)-HA (full symbols, black and red respectively) and the (*P*)-HA (empty symbols, black and red respectively). The color code is the same as in Figure 5.

The interaction between the double-stranded DNA and chiral helicene enantiopure compounds in 1:120 stoichiometry was then considered. Starting from initial trial geometries in a simulation cell as reported in Figure S2 (see Panel b), after the MD run and energy minimizations the optimized geometries are reported in Figure 7 for the (*M*)-HA and (*P*)-HA respectively on the left (Panel a) and on the right (Panel b).

Considering both the (*M*)-HA and the (*P*)-HA enantiomers at a high concentration at the end of the MD run, a similar stability of the system was calculated, as also found before at low concentration, as shown by the interaction energy calculated during MD run (central Panel in Figure 7). However, in this case the helicene molecules self-aggregate on the DNA surface when a smaller number of molecules were adsorbed on the major groove of DNA displaying a helical ordering induced by the adsorption on the helical structure of DNA. Following the animation of the MD run (see animation file reported below Figure S2 in SI), initially a layer of helicene molecules is adsorbed onto the DNA surface within 5 ns, parallel to a decrease more accentuated for the (*P*)-HA enantiomer in the potential energy and the van der Waals contribution as indicated by empty symbols, black and red respectively, in the central panel of Figure 7. The (*P*)-HA enantiomer kinetically adsorbed faster than the (*M*)-HA molecule. Therefore the aggregation process of the helicenes took place. Double-stranded DNA became like a nucleation center for hydrophobic aggregate formation.

It is important to study the adsorption on the DNA surface and also the self-aggregation process of chiral molecules. It is interesting to compare these theoretical results with experimental data in the literature. Studying the *N*-methyl-Aza[5]helicenium salts Latterini et al. explain that in the external region of cells the helicene dye was present in the monomeric form, which give rise to a short wavelength emission (580–620 nm), while in the cytoplasm the helicenium units form aggregates giving rise to shorter wavelength emission (500–530 nm) [48]. Probably the different concentration of helicenes in the outer region of cells and in the cytoplasm is the reason why the emission occurs at a shorter wavelength, then showing different colors in the confocal fluorescence images of the Hude fibroblast cells in which the molecules of helicene dye were able to penetrate inside cells. In particular the high affinity of helicenium salts already observed for DNA could suggest that the helicenium unit in the monomeric form were localized in the nuclear region of the cells, while the emission from helicenium aggregates were due to the reduced solubilization of the salt in the cytoplasm or, after this theoretical study, it will be possible that the concentration was not so high but at lower concentrations the helicene molecules could kinetically start the aggregation process due to the nucleation center on the chiral surface of the DNA double-stranded.

**Figure 8:**
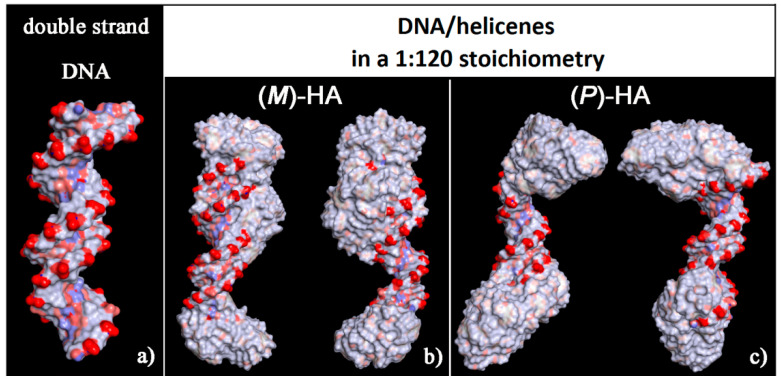
Solvent-accessible surface area colored by the atom charge for the optimized geometries of double-stranded DNA as data from Protein Data Bank on the left (**a**), of the (*M*)-HA (**b**) and (*P*)-HA on the right (**c**) after energy minimization of the conformation assumed by the system at the end of the MD run considering enantiopure compounds in a 1:120 stoichiometry. The color code is the same as in Figure 6.

The solvent-accessible surface area for optimized geometries after the MD run was reported in Figure 8. These new non-covalent DNA/helicene complexes exhibited different charge exposed to the biological environment than the native double-stranded DNA fragment. In fact, the molecule of helicene adsorbed and aggregated on the chiral structure of DNA partially hid the regular chiral arrangement of the phosphate groups.

### 3.4. DNA/Chiral 5-Aza[5]helicenes Interaction in Racemic Mixtures at Two Different Concentrations

**Figure 9:**
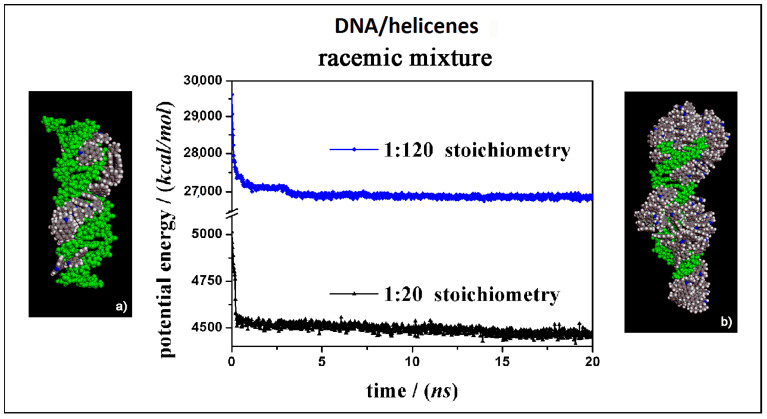
Optimized geometries of (*M*)-HA and (*P*)-HA in a racemic mixtures at low concentration on the left (**a**) and at high concentration on the right (**b**) after energy minimization of the conformation assumed by the system at the end of the MD run considering racemic mixtures in 1:20 and 1:120 stoichiometry respectively. In the central panel the potential energy calculated during MD run lasting 20 ns for racemic mixture in 1:20 stoichiometry (black symbols) and in 1:120 stoichiometry (blue symbols) respectively. The color code is the same as in Figure 5.

In this section the theoretical results on the interaction of the (*M*)-5-Aza[5]helicene and (*P*)-5-Aza[5]helicene molecules in racemic mixtures interacting with the external surface of the chiral DNA at two different concentrations, a low and a high concentration, are reported (see Figure 9).

It is curious to note that considering racemic mixtures at a low concentration (Panel a in Figure 9) that all the molecules were adsorbed on the external surface in particular in the major groove, and only very few molecules were adsorbed on the first hydrophobic helicene layer because of π–π interactions between aromatic rings, indicating that the racemic mixture favored the interaction with the DNA fragment. Even at high concentration (Panel b in Figure 9), the molecule layer in the major groove was adsorbed first, and then the aggregation of helicene molecules took place. Following the animation of the MD run (see animation files reported below Figure S2 in SI), initially a layer of helicene molecules is adsorbed onto the DNA surface within 5 ns both at 1:20 stoichiometry and in 1:120 stoichiometry, parallel to a decrease in the potential energy a as indicated in the central panel of Figure 9. In the competitive adsorption the (*P*)-HA enantiomer molecules kinetically adsorbs faster than the (*M*)-HA molecule. In the literature even Kel et al. reported that considering chiral cationic [4]helicene derivatives, which differ by their substituents and which show chiral selectivity in binding to double-stranded DNA, at a given concentration, the binding affinity measured with the racemic mixture is not the average of those of the enantiomeric (*M*)- and (*P*)-[4]helicene studied. For some [4]helicene binding with the racemic mixtures is stronger than with the enantiopure compound [21].

**Figure 10:**
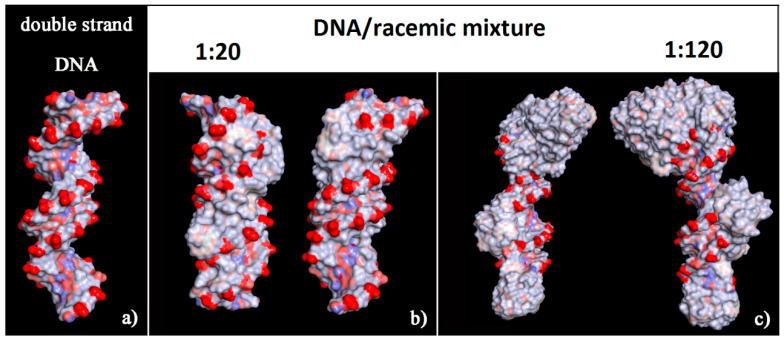
Solvent-accessible surface area colored by the charge of atoms for optimized geometries of double-stranded DNA as data from Protein Data Bank on the left (**a**), of the final optimized geometries of racemic mixtures at low concentration with (*M*)-HA/(*P*)-HA in a 1:20 stoichiometry in panel (**b**), and at high concentration with (*M*)-HA/(*P*)-HA in a 1:120 stoichiometry on the right in panel (**c**). The color code is the same as in Figure 6.

The surface of this complex modified the hydrophilic surface usually exposed to the biological environment by the double-stranded DNA with a number of hydrophobic helicenes that reproduce the helical shape of DNA (see the solvent-accessible surface area colored by the charge of atoms in Figure 10). In particular, the chiral molecules in contact with the major groove were the (*P*)-helicene enantiomers, as reported in Figure S3 (see the blue (*P*)-HA molecules on the left in panel a, and the top view of all helicenes adsorbed on the right in panel b). During the MD simulation time at room temperature the isomerization of these 5-Aza[5]helicenes was not observed (see Table 2), as it can be seen from the comparison with the Θ values reported in Table 1. Therefore, some changes were observed in the value of the Θ dihedral angles of two enantiomers adsorbed near the minor groove and the major groove of the DNA fragment with respect to the Θ values calculated for the isolated single molecule of [5]helicenes.

At a high concentration the adsorption process in the minor groove and preferentially in the major groove and afterwards the self-aggregation process of the helicene molecules took place. In the racemic mixtures a more ordered adsorption process was observed at low concentration and with aggregates on the first adsorbed helicene layer adhering stably during the MD run.

In this final case, it is important to underline that following the animation of the MD simulation performed also considering the racemic mixtures, the first step of chiral interaction on the DNA surface is the adsorption process of the first helicene layer preferentially in the major groove, and afterwards, at a high concentration, the aggregation forming well-ordered and larger aggregates well adhered to the DNA structure (see animation files reported below Figure S2 in SI). Again, even in the racemic mixture at a high concentration the non-covalently coated double-stranded DNA becomes like a nucleation center for the formation of hydrophobic aggregates, even if the concentration of helicene is not as high as to kinetically induce precipitation. Interestingly, in the competitive adsorption the (*P*)-HA enantiomers adsorbed faster and better on the chiral structure of the helical DNA than the enantiopure compound. Using the MM and MD theoretical methods preferential adsorption of one of two enantiomer molecules during the competitive adsorption in the racemic mixture can be investigated, and these methods can provide interesting results.

## 4. Conclusions

The MM and MD simulations are useful tool to study the adsorption process on the DNA surface of chiral helicenes to better understand the possible influence of intrinsic chirality and structural selectivity. At the atomistic level, the formation, structure and stability of the layer of chiral molecules physisorbed on the outer surface of double-stranded B-DNA was investigated considering enantiopure compounds or racemic mixtures at different enantiomer concentrations. With these methods, in addition to the effect of the surface chemistry exposed by the DNA surface, the influence of the nanoscale topography of this particular chiral structure was atomistically considered. Following the kinetics of the adsorption process during the MD runs, at first a helicene layer adsorbed on DNA surface was obtained, then nanoaggregates were formed by adsorbing on the first layer. The adsorption process of chiral hydrophobic molecules on the DNA could change the hydrophilic nature and it could partially modify its chirality exposed to the biological environment, with consequences for mechanisms involving DNA.

It was found that DNA became like a nucleation center for the formation of hydrophobic helicene aggregates inducing a significant change in the spectroscopic properties. Interestingly, in the competitive adsorption process, considering racemic mixtures, the (*P*)-HA enantiomer adsorbed quickly and following the chiral structure of the helical DNA with respect to (*M*)-HA enantiomers, as in experiments in which the racemic mixture exhibited more favorable affinity binding to DNA. Further investigations are already in progress about the protonated *N*-methyl-5-Aza[5]helicene enantiomers in order to understand their spontaneous aggregation in water and the adsorption process on the DNA surface, relevant for the optoelectronic properties of new nano-devices and in the therapeutic field and biomedical applications. Furthermore, it is interesting to point out that these molecules can be a potential next-generation of nanoparticle useful for targeted therapeutic delivery interacting with hydrophobic poorly soluble drugs.

## Figures and Tables

**Table 1:** Values of the interaction energy, *E_int_*, in kJ/mol calculated in the optimized geometries assumed at the end of MD run performed by two enantiomers studied for two different final geometries and the Θ values.

Site of Interaction	(*M*)-HA *E_int_* (kJ/mol)	(*P*)-HA *E_int_* (kJ/mol)	Θ Value (°)
Minor groove	−176.4	−181.0	−31.055°
Major groove	−166.0	−186.1	30.887°

**Table 2:** The values of Θ dihedral angles in the optimized geometries obtained at the end of the MD run considering the enantiopure compounds (*M*)-HA (negative Θ values in italics) and (*P*)-HA (positive Θ values) and the racemic mixture at a low concentration.

(*M*)-HA Θ Value (°)	(*P*)-HA Θ Value (°)	Racemic Mixture Θ Value (°)
*−34.845*	28.906	*−33.509*
*−34.042*	32.411	29.600
*−34.097*	30.561	29.335
*−34.309*	30.143	*−35.281*
*−34.571*	29.894	*−32.191*
*−29.006*	32.999	*−29.811*
*−30.403*	30.907	*−28.395*
*−35.449*	33.004	*−31.265*
*−27.265*	33.139	27.465
*−35.485*	35.388	30.308
*−29.408*	30.426	34.277
*−31.562*	29.240	30.720
*−32.144*	29.051	*−32.220*
*−31.986*	32.635	*−31.172*
*−32.355*	31.983	*−29.936*
*−32.067*	29.356	31.234
*−29.843*	29.356	29.065
*−31.724*	34.295	30.204
*−28.727*	32.277	*−33.123*
*−28.275*	30.206	32.905

## Supplementary Materials

Supplementary materials can be found at https://www.mdpi.com/1996-1944/13/21/5031/s1, Figure S1: Initial two different non-optimized geometries considered for the (M)-5-aza[5]helicene molecules on the left near the DNA minor groove (panel a), on the right near DNA major groove (panel b). The same geometries not reported here are also considered for the (P)-5-aza[5]helicene enantiomer. The carbon atoms are in grey, the nitrogen’s in blue, the oxygens in red, the phosphorus in pink and hydrogens in white, Figure S2: Initial non optimized two different geometries considered for the (*M*)-5-aza[5]helicene on the left (panel a) considering a small concentration (DNA/helicene in 1:20 stoichiometry), on the right considering a larger concentration (DNA/helicene in 1:120 stoichiometry). The same geometries are considered also for the (*P*)-5-aza[5]helicene enantiomer. In the panel b the DNA fragment is colored in green. The color code is the same as in Figure S1, Figure S3: Side view and top view of the optimized geometry of the racemic mixture of twenty enantiomers considering ten molecules of (M)-5-aza[5]helicene reported in red (Θ < 0) end ten molecules of (P)-5-aza[5]helicene reported in blue (Θ > 0) in the side view. In top view the color code is: carbon atoms in grey, nitrogen atoms in blue. The DNA structure and hydrogen atoms are omitted for clarity.

## Abbreviations

B-DNA	right-handed double-helical structure of DNA
Z-DNA	a different left-handed double-helical structure of DNA
MM	Molecular Mechanics
MD	Molecular Dynamics
NVT	Number of particles, volume and temperature constant during the MD run
NP	nanoparticle
(*M*)-HA	(*M*)-5-Aza[5]helicene
(*P*)-HA	(*P*)-5-Aza[5]helicene
PBC	Periodic boundary conditions
LD	linear dichroism
CD	circular dichroism
