# Supplementary material for: Adsorption of Chiral [5]-Aza[5]helicenes on DNA Can Modify Its Hydrophilicity and Affect Its Chiral Architecture: A Molecular Dynamics Study
Source: Materials (Basel). 2020 Nov 7;13(21):5031. doi: 10.3390/ma13215031 (PMC7664699; doi:10.3390/ma13215031)
Supplement: Supplementary file 1 [file materials-13-05031-s001.pdf]

materials

Supplementary

Adsorption of Chiral [5]-Aza[5]helicenes on DNA Can Modify Its Hydrophilicity and Affect Its Chiral Architecture: A Molecular Dynamics Study

Giuseppina Raffaini [1,2,*], Andrea Mele [1] and Tullio Caronna [3]

[1] Department of Chemistry, Materials and Chemical Engineering "Giulio Natta", Politecnico di Milano, Piazza L. Da Vinci 32, 20131 Milano, Italy; andrea.mele@polimi.it

[2] INSTM, National Consortium of Materials Science and Technology, Local Unit Politecnico di Milano, 20131 Milano, Italy

[3] Dipartimento di Ingegneria e Scienze Applicate, Università degli Studi di Bergamo, 24044 Bergamo, Italy; tullio.caronna@fastwebnet.it

* Correspondence: giuseppina.raffaini@polimi.it

Received: 14 September 2020; Accepted: 4 November 2020; Published: 7 November 2020

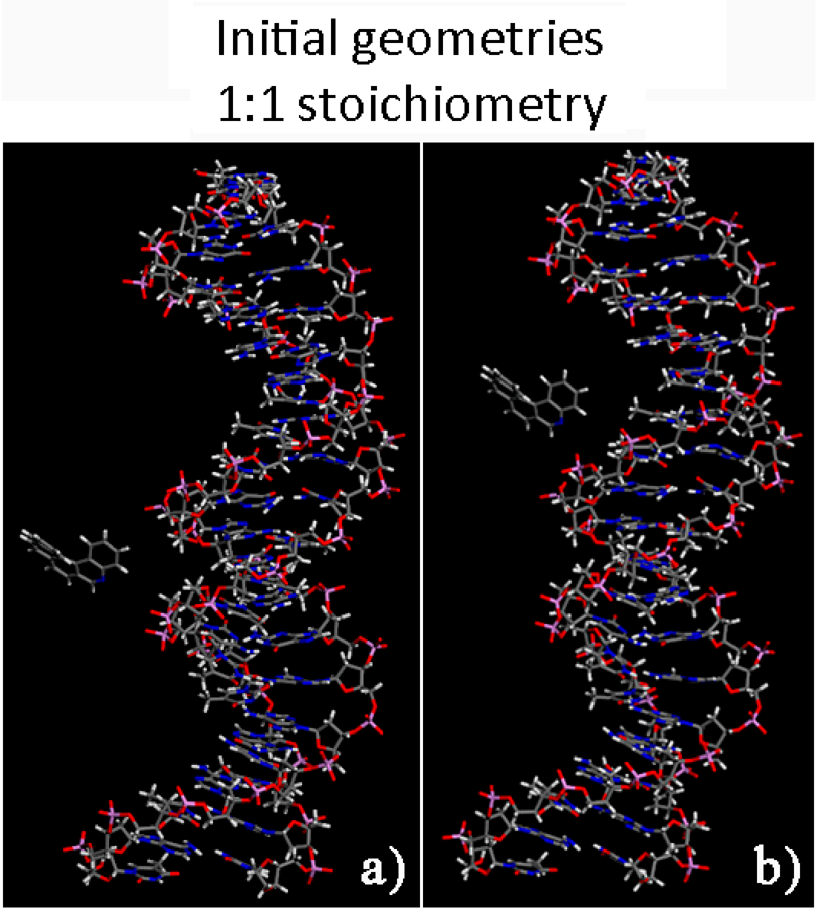

Figure S1. Initial two different non-optimized geometries considered for the (M)-5-aza[5]helicene molecules on the left near the DNA minor groove (panel a), on the right near DNA major groove (panel b). The same geometries not reported here are also considered for the (P)-5-aza[5]helicene enantiomer. The carbon atoms are in grey, the nitrogen's in blue, the oxygens in red, the phosphorus in pink and hydrogens in white.

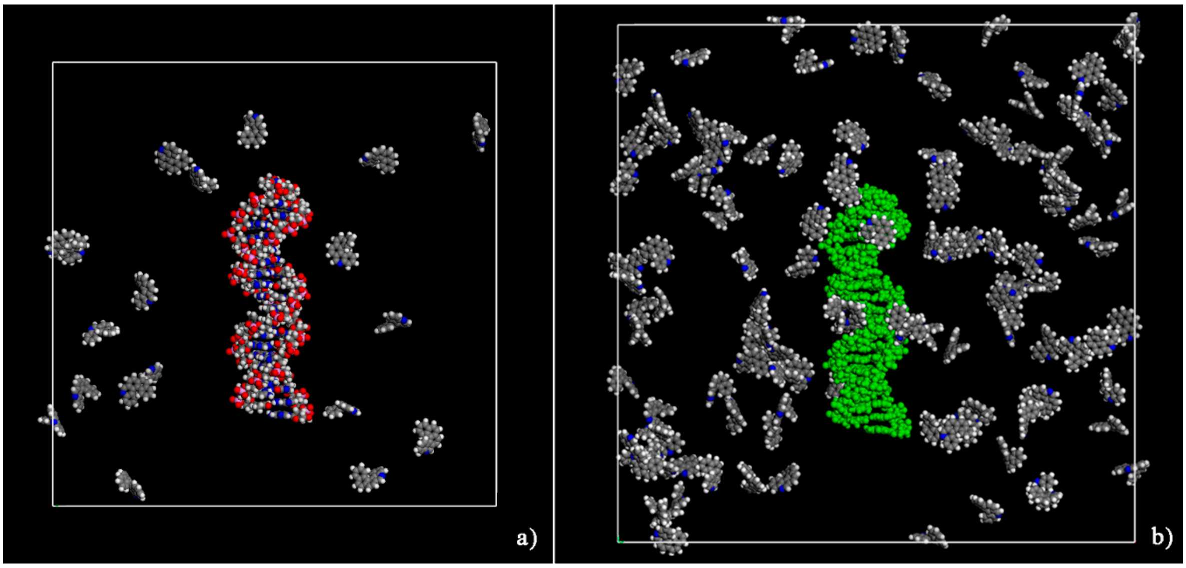

Figure S2. Initial non optimized two different geometries considered for the (*M*)-5-aza[5]helicene on the left (panel a) considering a small concentration (DNA/helicene in 1:20 stoichiometry), on the right considering a larger concentration (DNA/helicene in 1:120 stoichiometry). The same geometries are considered also for the (*P*)-5-aza[5]helicene enantiomer. In the panel b the DNA fragment is colored in green. The color code is the same as in Figure S1.

dyn DNA (M)-HA 20ns side view.avi
dyn DNA (M)-HA 20ns top view.avi
dynamics DNA (P)-HA 20ns side view.avi
DYN DNA (M)-HA 120ns side view.avi
DYN DNA (M)-HA 120ns top view.avi
DYNAMICS DNA (P)-HA 120ns side view.avi
dyn RACEMIC MIX 10(M)-HA 10(P)-HA 20ns.avi
dyn RACEMIC MIX 60(M)-HA 60(P)-HA 20ns.avi

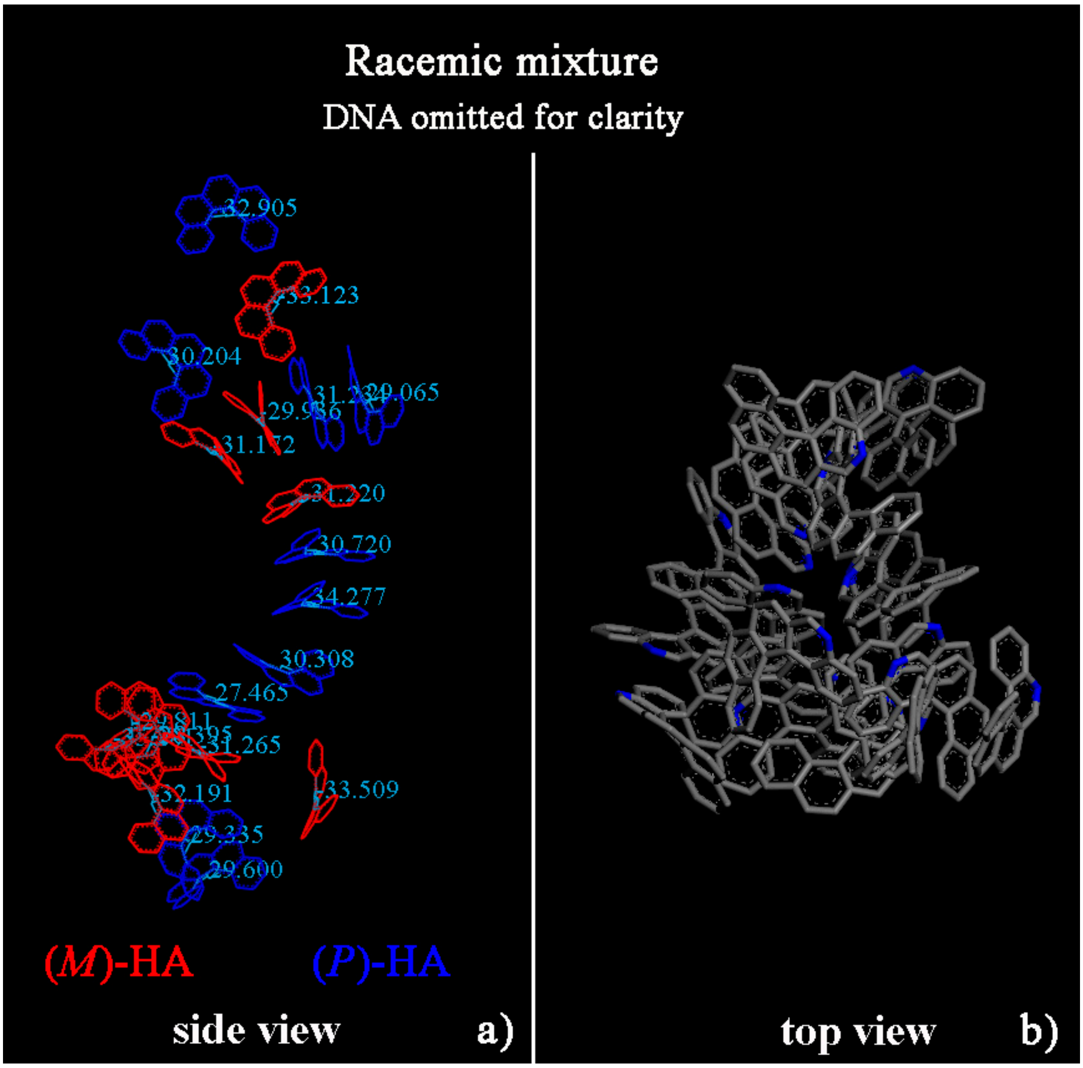

Figure S3. Side view and top view of the optimized geometry of the racemic mixture of twenty enantiomers considering ten molecules of (M)-5-aza[5]helicene reported in red (Θ < 0) end ten molecules of (P)-5-aza[5]helicene reported in blue (Θ > 0) in the side view. In top view the color code is: carbon atoms in grey, nitrogen atoms in blue. The DNA structure and hydrogen atoms are omitted for clarity.

© 2020 by the authors. Licensee MDPI, Basel, Switzerland. This article is an open access article distributed under the terms and conditions of the Creative Commons Attribution (CC BY) license (http://creativecommons.org/licenses/by/4.0/).
